# Postpartum depression and perceived stress among Tunisian parturient

**DOI:** 10.1192/j.eurpsy.2021.1837

**Published:** 2021-08-13

**Authors:** N. Messedi, L. Aribi, W. Bouattour, F. Khanfir, F. Charfeddine, K. Chaaben, J. Aloulou

**Affiliations:** 1 Psychiatry, Hedi Chaker University hospital, sfax, Tunisia; 2 Gynecology, Hedi Chaker University hospital, sfax, Tunisia

**Keywords:** parturient, post partum, Depression, stress

## Abstract

**Introduction:**

The postpartum depressions (PPD), rank first postpartum complications and therefore pose a public health problem by their frequencies and their adverse consequences.

**Objectives:**

To detect the depression among a Tunisian parturient, to evaluate their perceived stress and to study the link between these entities

**Methods:**

A cross-sectional, analytical study of 40 first week postpartum women hospitalized in the gynecology department in Hedi Cheker hospital in Sfax-Tunisia, during the month of September 2019. We used the Arab version of Edinburgh Postnatal Depression Scale (EPDS) and the Cohen perceived stress scale (PSS).

**Results:**

The average age of the participants was 31.07 years old. The Parturient have a rural origin in 62.5% of cases, they have a secondary school level in 52.5% of cases. There were exaggerated sympathetic signs in 52.5% of the cases. An organic pathologies were present during pregnancy in 47.5%. The postpartum period was simple in 77.5% of cases. For the post-natal period, 90% of parturient were going to receive help of a family member. EPDS: the average score was5.35 and the risk of developing a PPD was 20%. PSS we found that life represents a perpetual threat in 27% of cases. The factors correlated with the PPD were: a high level of perceived stress (p < 0.00) and organic pathology during pregnancy (p=0.02).

**Conclusions:**

Our study shows that the risk of postpartum depression is high among Tunisian parturient and it is associated with high level of stress, because of this a precocious screening is necessary.

**Disclosure:**

No significant relationships.

